# Incidence and risk factors of active carbapenem-resistant enterobacteriaceae surveillance in hematology patients: a propensity score matching study

**DOI:** 10.3389/fmicb.2025.1561587

**Published:** 2025-07-16

**Authors:** Shaozhen Chen, Jixin Fan, Tingting Xiao, Jinhua Ren, Haojie Zhu, Hui Kong, Dabing Chen, Jingjing Xu, Chenjing Ye, Jiaqi Sun, Caidong Hu, Xiaoyun Zheng, Jing Li, Xiaozhu Yang, Zhizhe Chen, Jianda Hu, Ting Yang

**Affiliations:** ^1^The Second Department of Hematology, National Regional Medical Center, Binhai Campus of the First Affiliated Hospital, Fujian Medical University, Fuzhou, China; ^2^The Second Department of Hematology, The First Affiliated Hospital, Fujian Medical University, Fuzhou, China; ^3^Fujian Medical University Cancer Hospital, Fujian Cancer Hospital, Fuzhou, China; ^4^Department of Hematology, Fujian Institute of Hematology, Fujian Provincial Key Laboratory of Hematology, Fujian Medical University Union Hospital, Fuzhou, China; ^5^Institute of Precision Medicine, Fujian Medical University, Fuzhou, China; ^6^Jinjiang Municipal Hospital (Shanghai Sixth People's Hospital Fujian), Quanzhou, China; ^7^The Second Affiliated Hospital of Fujian Medical University, Quanzhou, China

**Keywords:** Carbapenem-resistant Enterobacteriaceae, hematological diseases, incidence, risk factor, surveillance

## Abstract

**Introduction:**

Carbapenem-resistant Enterobacteriaceae (CRE) infections pose a significant threat to hematological patients, contributing to high mortality rates. This retrospective study evaluated the incidence, risk factors, and patient outcomes associated with active CRE surveillance in the hematology department.

**Methods:**

The study identified 23,832 hematological patients between 2019 and 2021. Propensity score matching was used to align underlying diseases and admission times in a 1:1:1 ratio across three groups: detected CRE, undetected CRE, and non-active CRE surveillance. The positivity rate of active CRE surveillance was 2.1% (141/6,735), with an incidence of 4.8% (85/1,789) among patients who underwent active CRE surveillance.

**Results:**

The distribution of the 141 isolates was as follows: *Klebsiella pneumoniae* (66.7%), *Escherichia coli* (22.6%), and others (10.7%). Independent risk factors associated with a positive result for active CRE surveillance included hematopoietic stem cell transplantation, hospital length of stay (LOS) ≥ 18 days, use of central venous catheters, steroid treatment within the past 3 months, antibiotic exposure (ß-lactam/ß-lactamase inhibitor, Echinocandins) within the last month, perianal skin ulceration within the previous 3 days, albumin < 33.4 g/L, and neutropenia lasting ≥ 7 days. In the detected CRE group, 26.5% of patients developed a CRE infection. Cox regression analysis identified diarrhea within 3 days prior to active CRE surveillance and interleukin-6 levels ≥ 39.35 pg./mL within 24 h of CRE surveillance as independent predictors of 90-day mortality. *Klebsiella pneumoniae* and *Escherichia coli* were the predominant pathogens identified in active CRE surveillance.

**Discussion:**

The incidence of CRE infection was notably higher in the detected CRE group. Our study provides real-world evidence on the role of active CRE surveillance in survival outcomes, especially in regions like China, where CRE infections are highly prevalent. The findings suggest that active CRE surveillance could serve as an early indicator of 90-day mortality in hematology patients and should be considered for routine implementation in this population.

## 1 Introduction

Carbapenem-resistant Enterobacteriaceae (CRE) colonization and infection represent a critical and escalating global public health threat. An analysis estimated that approximately 2.91 million people worldwide die from bloodstream infections each year, with around 392,000 deaths attributed to carbapenem resistance (Zha et al., 2025). In China, CRE carriage among hospitalized patients is increasingly common, with multicenter studies showing intestinal colonization rates around 8% in adults with hematologic diseases, and rates as high as 16% in intensive care and hematology wards (Yi and Kim, 2021; Hu et al., 2024). Notably, a substantial proportion of colonized patients progress to infection, a Chinese cohort found that 25.9% of CRE-colonized patients developed systemic CRE infections (Xiao et al., 2024). Clinically, CRE infection is associated with limited therapeutic options and high mortality, with multicenter studies reporting 30-day mortality rates for CRE bloodstream infections of approximately 50%—significantly higher than for carbapenem-susceptible strains (Baek et al., 2024). Long-term hospitalized patients serve as key reservoirs, and although not all colonized individuals develop infection, asymptomatic carriage greatly increases the risk of subsequent disease and nosocomial transmission.

Given these challenges, active CRE screening is considered a cornerstone strategy for containment. Robust evidence from Chinese hospitals shows that universal admission screening combined with pre-emptive contact isolation in high-risk wards can dramatically reduce hospital-acquired CRE infections. A multicenter study reported that, in neonatal intensive care units, this approach lowered CRE infection rates from 1.96 to 0.63%, and in general neonatal wards from 0.57 to 0.30% (Yin et al., 2020). Similarly, in adult emergency intensive care units, rapid molecular screening plus bundled infection control interventions reduced overall CRE incidence from 5.24 to 3.48% (Zhou et al., 2023). These findings underscore that sustained, high-compliance active surveillance and comprehensive prevention strategies are highly effective for curbing CRE transmission, especially among immunocompromised, critically ill, and long-stay patients (Yang et al., 2023).

Patients with hematological diseases (HDs) are especially vulnerable to multidrug-resistant (MDR) infections, notably CRE. Multicenter studies have shown that CRE colonization or infection is independently associated with more than a threefold increase in 30-day mortality among patients with acute leukemia receiving intensive chemotherapy (Ballo et al., 2019). Accordingly, this study aims to evaluate the impact of active CRE surveillance in the hematology ward and to identify mortality-associated risk factors by comparing patients who underwent active surveillance with those who did not.

## 2 Materials and methods

### 2.1 Study design and patient selection

**Figure 1:**
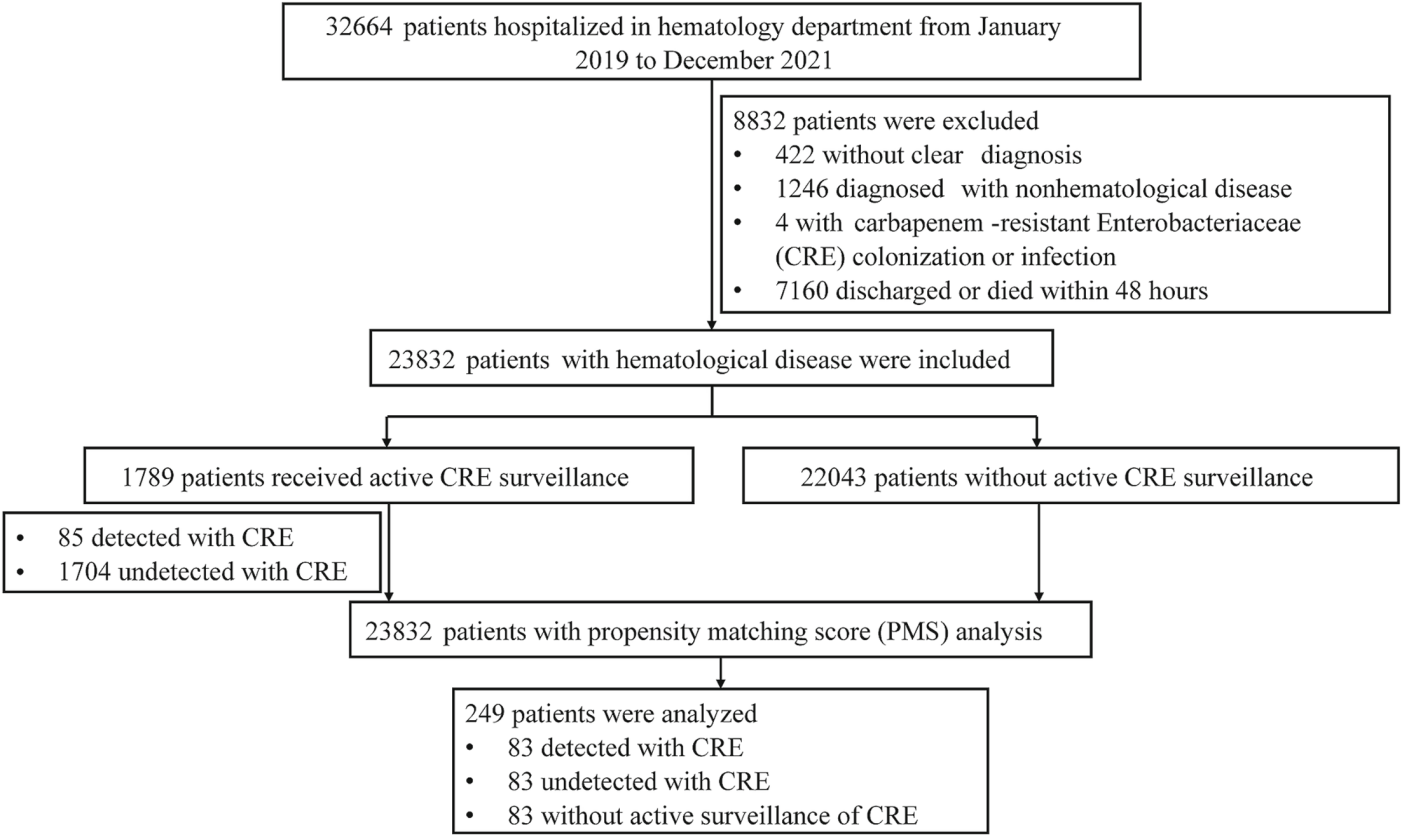
Flow chart of study selection process.

This study included patients with hematological diseases, who were followed at the Department of Hematology at Fujian Medical University Union Hospital (FMUH), a large general teaching hospital in Fuzhou, China, between January 1, 2019, and December 31, 2021. The medical records of these patients were retrospectively reviewed. The inclusion criteria were: admission to the hematology department and a diagnosis of any hematological disease. Exclusion criteria included unclear diagnoses, non-hematological disease diagnoses, CRE colonization or infection, and discharge or death within 48 h. A total of 23,832 patients met the inclusion criteria, and 1,789 of these patients received active CRE surveillance. After Propensity Score Matching (PSM) based on types of hematological diseases and admission time, 249 patients were enrolled and divided into three groups (Table 1). Patients with a positive culture from active CRE surveillance were categorized to the “detected CRE” group, those with a negative culture as the “undetected CRE” group, and those who did not undergo active CRE surveillance as the “non-active CRE surveillance” group. The study flow chat is presented in Figure 1. For patients undergoing multiple active CRE surveillances, clinical data from the first positive result or the first surveillance during the same hospitalization were used. For patients without active CRE surveillance, clinical data from the time of admission during the same hospitalization were included. This study was approved by the ethics committee of Fujian Medical University Union Hospital, and written informed consent was obtained from all patients in accordance with the Helsinki Declaration.

**Table 1:** Group comparisons before and after propensity score matching (PSM) for patient’s characteristics.

Characteristics	Before PSM matching			*P*	After PSM matching			*P*
	Active CRE surveillance		Non-active CRE surveillance		Active CRE surveillance		Non-active CRE surveillance	
	Detected CRE (*n* = 85)	Undetected CRE (*n* = 1704)	(*n* = 22,043)		Detected CRE (*n* = 83)	Undetected CRE (*n* = 83)	(*n* = 83)	
Underlying disease				<0.001				1.0
AML	42(49.4)	675(39.6)	3,747(17.0)		42(50.6)	42(50.6)	42(50.6)	
ALL	14(16.5)	293(17.2)	4,250(19.3)		14(16.9)	14(16.9)	14(16.9)	
MDS	6 (7.0)	77(4.5)	761(3.5)		5(6.0)	5(6.0)	5(6.0)	
MM	7(8.2)	182(10.7)	2,168(9.8)		7(8.4)	7(8.4)	7(8.4)	
NHL	10(11.8)	336(19.7)	8,503(38.6)		10(12.0)	10(12.0)	10(12.0)	
HL	1(1.2)	14(0.8)	843(3.8)		1(1.2)	1(1.2)	1(1.2)	
AA	1(1.2)	67(3.9)	319(1.4)		1(1.2)	1(1.2)	1(1.2)	
Others	4(4.7)	60(3.5)	1,452(6.6)		3(3.6)	3(3.6)	3(3.6)	
Admission time, median (range), days	21(5, 37)	19(4, 40)	21(3, 39)	<0.001	21(5, 37)	21(4, 37)	21(5, 37)	0.97

Data are expressed as mean (range) or percent (%). Continuous and categorical variables were compared using Student’s t test (or Mann–Whitney test) and Chi-square test (or Fisher’s exact test), respectively. PSM, propensity score matching; CRE, carbapenem-resistant enterobacteriaceae; AML, acute myeloid leukemia; ALL, acute lymphoblastic leukemia; MDS, myelodysplastic syndromes; MM, multiple myeloma; NHL, non-Hodgkin’s lymphoma; HL, Hodgkin’s lymphoma; AA, aplastic anemia.

### 2.2 Active CRE surveillances and antimicrobial susceptibility

Stool, perianal and oropharyngeal swabs were collected within 48 h of admission. Routine follow-up screening was then performed once weekly for standard-risk hematology in-patients and twice weekly (every Monday and Thursday) for high-risk subgroups (allogeneic HSCT recipients, patients with expected neutropenia ≥ 7 days, or those in non-remission status) until discharge or death. Additional ‘on-demand’ screening was initiated if unexplained fever, diarrhea, or perianal ulceration developed. A total of 6,735 stool, perianal, or oropharyngeal swabs were collected from 1,789 patients and inoculated onto eosin methylene blue (EMB) agar plates with a 10 μg meropenem disk (Giani et al., 2012; Alizadeh et al., 2018). The plates were incubated at 35°C for 24 h, resulting in the identification of 141 CRE isolates using the Vitek-2 Compact System (BioMérieux, Marcy-l’Étoile, France). These isolates were stored at −80°C for antimicrobial susceptibility testing. Antimicrobial susceptibility was evaluated for the following antibiotics: Cefpodoxime, Cefixime, Cefoxitin, Cefazolin, Cefaclor, Ceftriaxone, Cefepime, Cefotaxime, Ceftazidime, Cefoperazone-sulbactam, Amoxicillin-clavulanic acid, Piperacillin-tazobactam, Ertapenem, Meropenem, Imipenem, Aztreonam, Compound Sulfamethoxazole, Gentamicin, Tobramycin, Amikacin, Levofloxacin, Norfloxacin, Moxifloxacin, Ciprofloxacin, Nitrofurantoin, Colistin, Minocycline, Doxycycline, Tigecycline, and Fosfomycin. Testing was performed using agar dilution and microdilution methods according to Clinical and Laboratory Standards Institute (CLSI) guidelines (Humphries et al., 2021). Quality control was ensured with *Escherichia coli* ATCC 25922 and *Klebsiella pneumoniae* ATCC 700603 strains, as recommended by CLSI guidelines.

### 2.3 Parameter definitions

CRE are a group of *Enterobacteriaceae* bacteria that exhibit resistance to at least one carbapenem antibiotic, defined by minimum inhibitory concentrations (MICs) of ≥ 4 μg/mL for doripenem, meropenem, or imipenem, or ≥ 2 μg/mL for ertapenem, or are documented to produce carbapenemase. For organisms with intrinsic resistance to imipenem (e.g., *Morganella morganii*, *Proteus* spp., *Providencia* spp.), resistance to carbapenems other than imipenem is required (Chen et al., 2024a). In our study, molecular methods (e.g., PCR detection of carbapenemase genes such as KPC, NDM, and OXA-48) were not employed, and thus CRE were identified solely based on phenotypic antimicrobial susceptibility testing.

Neutropenia was defined as an absolute neutrophil count (ANC) of ≤ 0.5 × 10^9^/L, or ≤ 1.0 × 10^9^/L with a predicted decline of ≤ 0.5 × 10^9^/L within 24–48 h (El Omri et al., 2024; Islas-Muñoz et al., 2024). MDR was defined as bacterial resistance to three or more classes of antibiotics (Thaden et al., 2017).

### 2.4 Statistical analysis

We performed one-to-one-to-one nearest-neighbor matching without replacement among the three groups (CRE-detected, CRE-undetected, no surveillance). Propensity scores were estimated using a multinomial logistic regression model including key clinical covariates. Matching was implemented with the ‘MatchIt’ package in R (version 4.1.0), using a caliper width of 0.2 times the standard deviation of the logit of the propensity score, as recommended (Austin, 2011). Continuous variables were expressed as medians with interquartile ranges, while categorical variables were presented as counts and percentages, differences in normally distributed continuous variables between groups were assessed using the Student’s t-test, whereas Mann–Whitney U tests was applied for non-normally distributed variables. Categorical variables were compared using the Chi-square test or Fisher’s exact test. Multivariate analysis was conducted using logistic regression models to identify risk factors associated with positive results in active CRE surveillance. Odds ratios (OR) with 95% confidence intervals (CI) were reported. For assessing risk factors for 90-day mortality in hematology patients, Cox proportional hazards regression was used, with hazard ratios (HR) and 95% CI reported. All statistical tests were two-sided, and a *p*-value < 0.05 was considered statistically significant. Data analyzes were preformed using SPSS version 26.0 (SPSS Inc., Chicago, IL).

## 3 Results

### 3.1 Characteristics of patients with hematological diseases

The characteristics of the patients in the entire cohort are summarized in Table 2. No significant differences in sex distribution were observed among the three groups (*p* = 0.754), and the median age was comparable (46 years *vs.* 47 years *vs.* 47 years). The prevalence of diabetes was also similar among the groups (14.5% *vs.* 20.5% *vs.* 13.3%). Notably, the hospital length of stay (LOS) was significantly longer in the detected CRE group compared to other groups (*p* < 0.001). Patients in the non-active CRE surveillance group had the highest remission rate and were the least likely to have undergone hematopoietic stem cell transplantation. Additionally, hospitalization costs were highest in the detected CRE group, followed by the undetected CRE group. The median follow-up times were 110, 268, and 270 days in the three groups, respectively.

**Table 2:** Characteristics of 249 patients after PSM analysis between 2019 and 2021.

Characteristics	Detected CRE (*n* = 83)	Undetected CRE (*n* = 83)	Non-active CRE surveillance (*n* = 83)	*P*
Sex				0.754
Female	33(39.8)	38(45.8)	36(43.4)	
Male	50(60.2)	45(54.2)	47(56.6)	
Age, years	46(2–84)	47(7–82)	47(1–83)	0.581
Diabetes mellitus				0.419
Absence	71(85.5)	66(79.5)	72(86.7)	
Presence	12(14.5)	17(20.5)	11(13.3)	
Length of stays, days	36(10–290)	27(2–103)	8(2–60)	<0.001
Receiving HSCT				<0.001
No	48(57.8)	48(57.8)	79(95.2)	
Yes	35(42.2)	35(42.2)	4(4.8)	
Length of diseases, months	6.0(0–72)	3.0(0–70)	3.0(0–79)	0.045
Disease status before CRE surveillance				<0.001
Remission	17(21.0)	26(31.3)	41(50.6)	
No remission	64(79.0)	57(68.7)	40(49.4)	
Number of previous chemotherapy	3.0(0–21)	2.0(0–22)	3.0(0–41)	0.021
Number of CRE active surveillance	4.0(1–30)	3.0(1–25)	0.0(0–0)	<0.001
Total hospital costs, Rmb	174213.6(6053.9–750298.2)	127072.6(6172.1–702680.7)	15652.2(1677.7–364444.1)	<0.001
Follow-up period, days	110(10–1,128)	268(8–1,150)	270(4–1,019)	0.011

Data are expressed as mean (range) or percent (%). Continuous and categorical variables were compared using Student’s *t*-test (or Mann–Whitney test) and Chi-square test (or Fisher’s exact test), respectively. CRE, carbapenem-resistant enterobacteriaceae; AML, acute myeloid leukemia; ALL, acute lymphoblastic leukemia; MDS, myelodysplastic syndromes; MM, multiple myeloma; NHL, non-Hodgkin’s lymphoma; HL, Hodgkin’s lymphoma; AA, aplastic anemia; HSCT, hematopoietic stem cell transplantation.

### 3.2 Positive rate of active CRE surveillance and distribution of CRE strains

**Figure 2:**
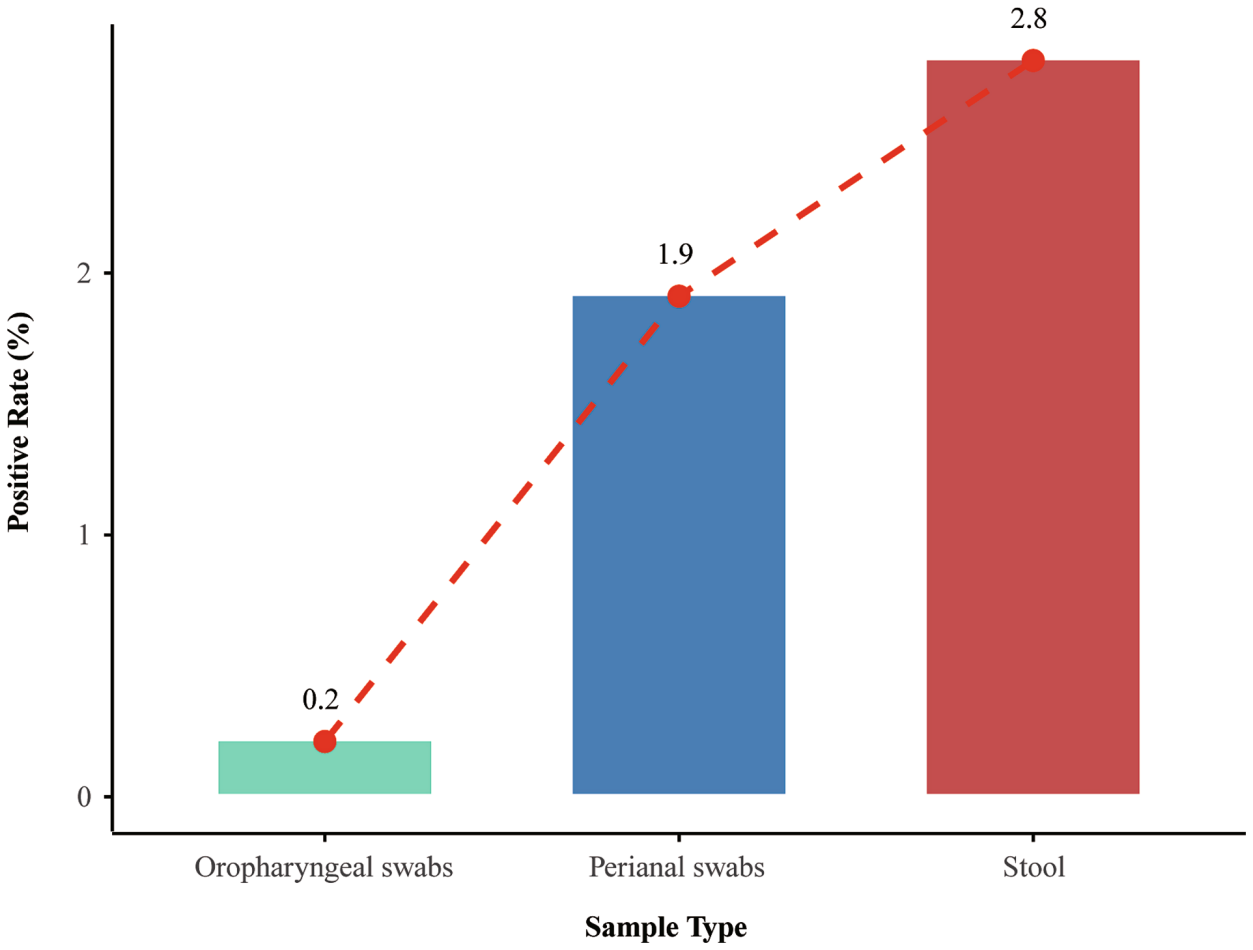
Positive rates of active CRE screening across 3,215 stool, perianal, and oropharyngeal swab samples collected from 304 patients with simultaneous testing.

A total of 6,735 samples from 1,789 patients underwent active CRE surveillance. The positivity rate of active CRE surveillance, based on stool, perianal, or oropharyngeal swabs, was approximately 2.1% (141/6,735). Among the patients, 4.8% (85/1,789) were detected with CRE, with the highest detection rate observed in 2019 (7.0%), while the rates in 2020 and 2021 were comparatively lower (Seen in Supplementary Figure 1). Among the 3,215 samples provided by 304 patients who underwent simultaneous testing of stool, perianal swabs, and oropharyngeal swabs, stool samples exhibited the highest positivity rate at 2.8%, followed by perianal swabs and oropharyngeal swabs (Figure 2). Pathogens isolated from stool or perianal swabs from the same patient were identified as originating from the same source. Notably, 48 strains among the total 141 were excluded as they originated from the same source. Of the 93 strains from different sources, the most frequently identified pathogens were *Klebsiella pneumoniae* (66.7%), followed by *Escherichia coli* (22.6%), *Enterobacter cloacae* (6.5%), and other CRE strains (Seen in Supplementary Table1).

### 3.3 Antimicrobial resistance of major CRE pathogens

Our antimicrobial susceptibility data for the 93 pathogens showed that CRE strains were nearly 100% resistant to cephalosporins and *β*-lactamase inhibitor combinations, although they displayed minimal resistance to cefepime, a fourth-generation cephalosporin. Notably, CRE strains were generally more susceptible to colistin (63.6%), moxifloxacin (71.4%), tigecycline (78.2%), and amikacin (63.7%).

Among the *Klebsiella pneumoniae* isolates, 100.0% were resistant to cephalosporins, *β*-lactamase inhibitor combinations, and nitrofurantoin, except for cefepime. Over 90.0% of *K. pneumoniae* isolates demonstrated resistance to carbapenems, monobactams, levofloxacin, and ciprofloxacin. However, these isolates exhibited relatively low resistance rates to moxifloxacin (25.0%) and fosfomycin (20.0%). Interestingly, only 3.0% of *K. pneumoniae* isolates were resistant to tigecycline, and no resistance was observed to colistin.

Similarly, *Escherichia coli* isolates displayed high resistance to cephalosporins and β-lactamase inhibitor combinations. In contrast, only 33.3% of *E. coli* strains were resistant to norfloxacin and moxifloxacin. Notably, none of the *E. coli* strains exhibited resistance to fosfomycin, nitrofurantoin, colistin, or tigecycline (Table 3).

**Table 3:** Susceptibility of major CRE pathogens to different antibacterial drug.

Antibiotic	Total (*n* = 93, %)			CRKP (*n* = 62, %)			*E. coli* (*n* = 21, %)		
	R	I	S	R	I	S	R	I	S
Cephalosporins									
Cefpodoxime	100.0	0.0	0.0	100.0	0.0	0.0	100.0	0.0	0.0
Cefixime	100.0	0.0	0.0	100.0	0.0	0.0	100.0	0.0	0.0
Cefoxitin	100.0	0.0	0.0	100.0	0.0	0.0	100.0	0.0	0.0
Cefazolin	100.0	0.0	0.0	100.0	0.0	0.0	100.0	0.0	0.0
Cefaclor	100.0	0.0	0.0	100.0	0.0	0.0	100.0	0.0	0.0
Ceftriaxone	100.0	0.0	0.0	100.0	0.0	0.0	100.0	0.0	0.0
Cefepime	98.9	0.0	1.1	98.4	0.0	1.6	100.0	0.0	0.0
Cefotaxime	100.0	0.0	0.0	100.0	0.0	0.0	100.0	0.0	0.0
Ceftazidime	100.0	0.0	0.0	100.0	0.0	0.0	100.0	0.0	0.0
β-lactamase inhibitor combinations									
Cefoperazone-sulbactam	100.0	0.0	0.0	100.0	0.0	0.0	100.0	0.0	0.0
Amoxicillin-Clavulanic acid	100.0	0.0	0.0	100.0	0.0	0.0	100.0	0.0	0.0
Piperacillin/tazobactam	98.9	1.1	0.0	100.0	0.0	0.0	100.0	0.0	0.0
Carbapenems									
Ertapenem	100.0	0.0	0.0	100.0	0.0	0.0	100.0	0.0	0.0
Meropenem	97.0	0.0	3.0	94.7	0.0	5.3	100.0	0.0	0.0
Imipenem	92.0	4.5	3.5	94.7	3.5	1.8	81.0	9.5	9.5
Monobactams									
Aztreonam	90.3	0.0	9.7	95.2	0.0	4.8	81.0	0.0	19.0
Sulfonamide									
Compound sulfamethoxazole	86.0	0.0	14.0	83.9	0.0	16.1	90.5	0.0	9.5
Aminoglycosides									
Gentamicin	70.0	6.7	23.3	71.4	4.8	23.8	72.7	0.0	27.3
Tobramycin	62.6	13.2	24.2	63.3	10.0	26.7	62.0	19.0	19.0
Amikacin	35.2	1.1	63.7	42.6	1.6	55.8	14.3	0.0	85.7
Fluoroquinolones									
Levofloxacin	91.3	6.5	2.2	91.9	4.8	3.3	85.0	0.0	15.0
Norfloxacin	55.6	11.1	33.3	60.0	20.0	20.0	33.3	0.0	66.7
Moxifloxacin	28.6	0.0	71.4	25.0	0.0	75.0	33.3	0.0	66.7
Ciprofloxacin	94.4	1.1	4.5	95.0	1.7	3.3	89.5	0.00	10.5
Nitrofurans									
Nitrofurantoin	75.0	0.0	25.0	100.0	0.0	0.0	0.0	0.0	100.0
Polypeptide antibiotics									
Colistin	9.1	27.3	63.6	0.0	16.7	83.3	0.0	50.0	50.0
Tetracyclines									
Minocycline	82.8	5.2	12.0	83.3	4.8	11.9	80.0	0.0	20.0
Doxycycline	84.8	6.1	9.1	80.0	10.0	10.0	100.0	0.0	0.0
Tigecycline	1.8	20.0	78.2	3.0	30.3	66.7	0.0	5.0	95.0
Phosphonic acid									
Fosfomycin	16.7	33.3	50.0	20.0	40.0	40.0	0.0	0.0	100.0

CRE, carbapenem-resistant enterobacteriaceae; CRKP, carbapenem-resistant *Klebsiella pneumoniae*; E. coli, *Escherichia coli*; S, sensitive to tested antibiotic; I, intermediate resistant to tested antibiotic; R, resistant to tested antibiotic.

### 3.4 Risk factors for isolation of CRE from hematological patients

Univariate analysis identified numerous factors significantly associated with CRE isolation among hematology patients. After adjustment in a multivariate logistic regression model, a subset of these factors remained independent risk factors for a positive CRE surveillance culture. Specifically, patients who underwent HSCT, had a prolonged hospital stay (LOS ≥ 18 days), had a central venous catheter in place, or received recent steroid therapy were at higher risk of CRE isolation. In addition, exposure to certain antimicrobial agents (*β*-lactam/β-lactamase inhibitor antibiotics and echinocandins), the presence of perianal skin ulceration, hypoalbuminemia (albumin < 33.4 g/L), and prolonged neutropenia (duration ≥ 7 days) were independently associated with an increased likelihood of CRE detection. These factors constituted the final multivariate model for CRE isolation. (Table 4; details in Supplementary Table 2).

**Table 4:** Univariate and multivariate logistic regression analysis of risk factors for isolation of CRE.

Variable	Univariable model		Multivariable model	
	OR (95%CI)	*P* value	OR (95%CI)	*P* value
Receiving HSCT				
No	1.0 (Ref)		1.0 (Ref)	
Yes	14.4(4.8–43.0)	<0.001	48.2 (4.9–474.8)	0.001
Hospital length of stays				
<18	1.0 (Ref)		1.0 (Ref)	
≥18	76.7 (24.6–239.1)	<0.001	23.7 (3.7–151.0)	0.001
Central venous catheter				
Absence	1.0 (Ref)		1.0 (Ref)	
Presence	5.7 (2.5–12.9)	<0.001	20.8 (3.4–127.2)	0.001
Exposure to steroid within 3 months				
No	1.0 (Ref)		1.0 (Ref)	
Yes	11.1 (5.4–23.2)	<0.001	4.5 (1.1–18.3)	0.037
ß-lactam/ß-lactamase inhibitor				
No	1.0 (Ref)		1.0 (Ref)	
Yes	32.0 (13.3–76.5)	<0.001	16.0 (2.9–88.1)	0.001
Echinocandins antifungal				
No	1.0 (Ref)		1.0 (Ref)	
Yes	21.2 (7.1–63.3)	<0.001	12.0 (1.3–115.7)	0.031
Perianal skin ulceration				
No	1.0 (Ref)		1.0 (Ref)	
Yes	10.4 (4.9–25.6)	<0.001	7.3 (3.8–16.4)	<0.001
Albumin, g/L				
≥33.4	1.0 (Ref)		1.0 (Ref)	
<33.4	8.2 (3.9–17.1)	<0.001	6.0 (1.1–33.0)	0.039
Duration of neutropenia prior to CRE active surveillance				
<7	1.0 (Ref)		1.0 (Ref)	
≥7	19.3 (6.5–57.5)	<0.001	38.5 (3.0–493.9)	0.005

HSCT, hematopoietic stem cell transplantation; MDR, multiple drug resistance.

### 3.5 Outcomes and risk factors for 90-day mortality in the patients receiving active CRE surveillance

**Figure 3:**
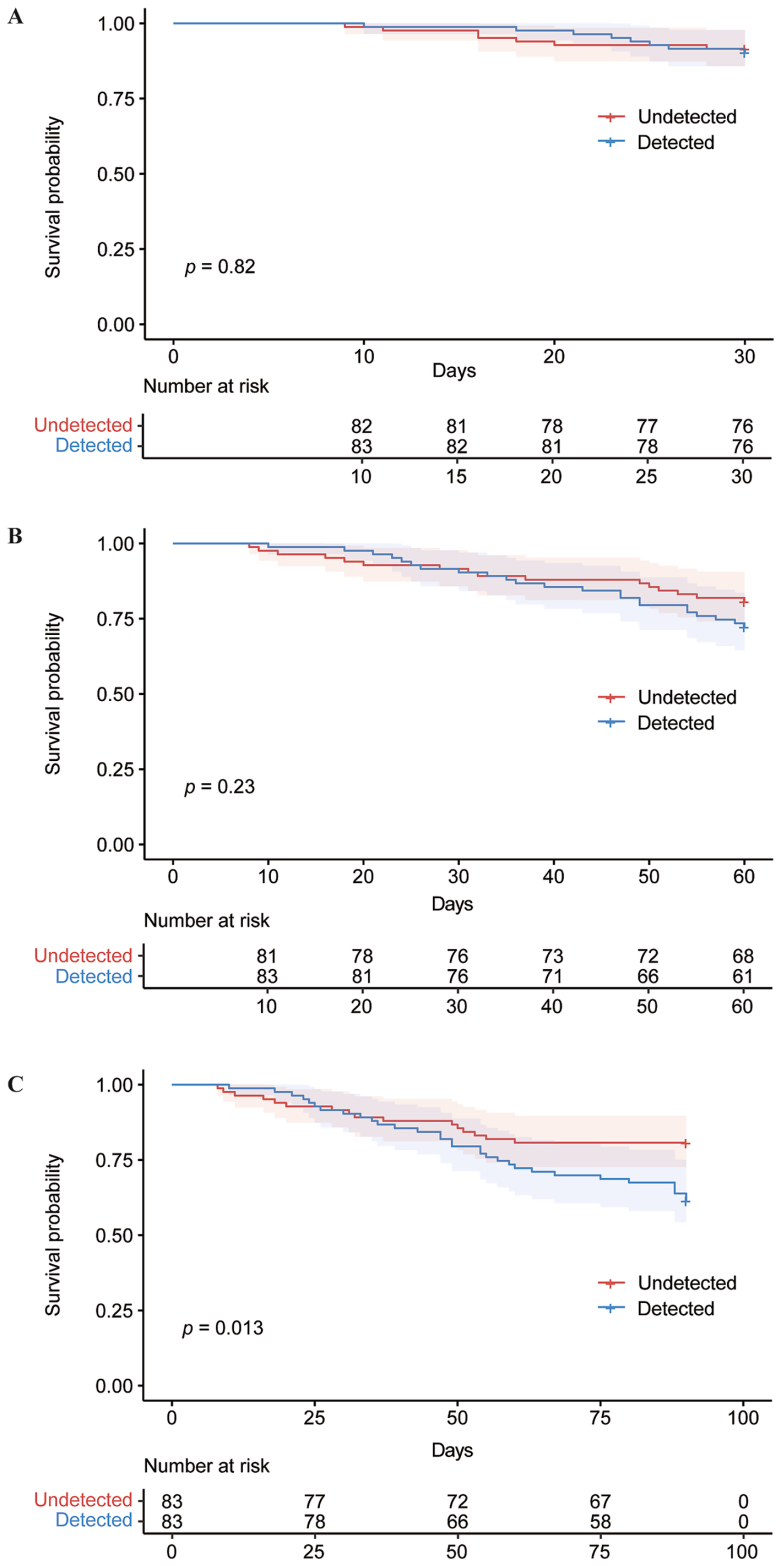
Survival analysis of patients in the Detected CRE and Undetected CRE groups using Kaplan–Meier curves. **(A)** Kaplan–Meier estimates of 30-day patient mortality for the Detected CRE and Undetected CRE groups. **(B)** Kaplan–Meier estimates of 60-day patient mortality for the Detected CRE and Undetected CRE groups. **(C)** Kaplan–Meier estimates of 90-day patient mortality for the Detected CRE and Undetected CRE groups.

There was no significant difference in the mortality rates at day 30 and day 60 between the detected and undetected CRE groups (Figures 3A,B). However, the detected CRE group exhibited a higher mortality rate at day 90 compared to the undetected group, as shown in Figure 3C. Among patients undergoing active CRE surveillance, univariate analysis showed that many clinical and laboratory factors were associated with higher 90-day mortality. In the multivariate Cox regression model, however, only two variables emerged as significant independent predictors of 90-day mortality. Specifically, patients who experienced diarrhea within the 3 days prior to CRE surveillance and those with an elevated interleukin-6 level (≥ 39.35 pg./mL) at the time of surveillance had a substantially higher risk of death by 90 days. (Table 5; details in Supplementary Table 3).

**Table 5:** Univariate and multivariate cox regression analysis of risk factors associated with 90-day mortality among patients detected CRE.

Variable	Univariable model		Multivariable model	
	HR (95%CI)	*P* value	HR (95%CI)	*P* value
Diarrhea				
No	1.0 (Ref)		1.0 (Ref)	
Yes	2.8 (1.4–5.5)	0.003	9.7 (1.2–81.8)	0.036
Interleukin-6, pg/mL				
<39.35	1.0 (Ref)		1.0 (Ref)	
≧39.35	2.6 (1.2–5.5)	0.014	7.8 (1.3–46.6)	0.025

CRE, carbapenem-resistant enterobacteriaceae.

### 3.6 Impact of CRE screening frequency on infection and survival outcomes

**Figure 4:**
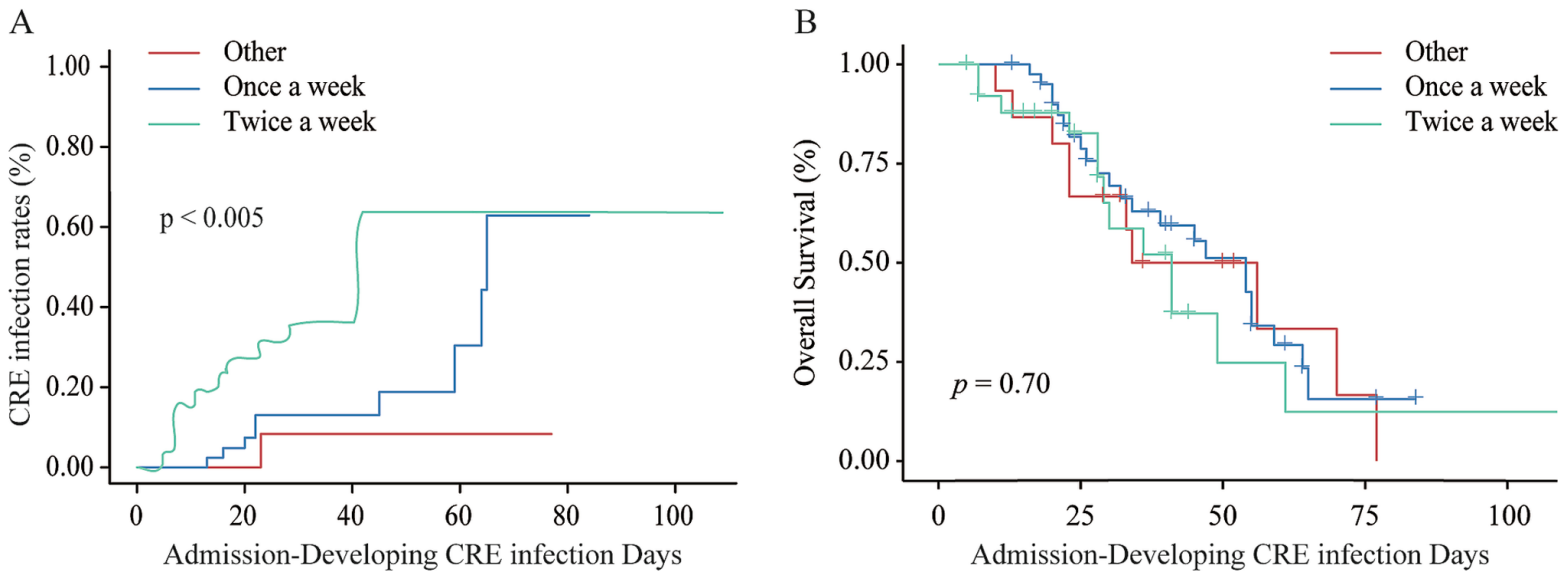
Impact of CRE screening frequency on infection rates and survival outcomes. **(A)** CRE infection rates between high and low screening frequencies in the detected CRE group. **(B)** Overall survival comparison between different CRE screening frequencies in the detected CRE group.

In the detected CRE group, 22 patients developed CRE infection, resulting in an infection rate of 26.5%. In contrast, only 1 patient in the undetected CRE group was infected, with an infection rate of 1.2%. Among patients in the detected CRE group, a higher screening frequency was significantly more effective in the early detection of CRE infections compared to a lower screening frequency (*p* = 0.005, Figure 4A). However, the frequency of CRE screening did not influence overall survival, as no statistically significant difference observed (*p* = 0.70, Figure 4B).

## 4 Discussion

CRE remains a global public health threat, particularly for immunocompromised patients such as those with HD and transplant recipients. Currently, treatment options for CRE are limited, with polymyxin, tigecycline, and fosfomycin as the primary therapies. Additionally, ceftazidime-avibactam has emerged as a common treatment option for CRE infections (Guo et al., 2023; Zeng et al., 2023). The prognosis for HD patients with CRE infections is generally poor (Lalaoui et al., 2020; Chen et al., 2024b), though combination therapies and newer drugs may provide improved outcomes. Understanding the epidemiological characteristics, drug resistance profiles, and mechanisms of antibiotic resistance in CRE is crucial for developing effective antimicrobial strategies for HD patients. In this study, we analyzed the distribution of CRE under active surveillance with *Klebsiella pneumoniae* and *Escherichia coli* being the predominant strains, together accounting for 89.3%. These findings align with previous studies (Satlin et al., 2014; Pouch and Satlin, 2017).

The primary resistance mechanisms in CRE are enzyme synthesis, efflux pumps, and porin mutations, with enzyme synthesis being the major mechanism. Enzymes involved in carbapenem resistance belong to three classes (Ambler class A, B, and D), including KPC, NDM, IMP, VIM, and OXA-48, among others (Wang et al., 2021). These *β*-lactamase confer resistance by hydrolyzing carbapenems and other β-lactam antimicrobials, collectively referred to as carbapenemases. In our analysis, resistance rates to fosfomycin, colistin, and tigecycline were determined to be 16.7, 9.1, and 1.8%, respectively. Interestingly, resistance to fosfomycin and tigecycline was almost exclusively associated with *E. coli*. Among 294 non-duplicated CRKP strains from two tertiary hospitals in China, 18.71% (55/294) exhibited fosfomycin resistance. Globally, reported resistance rates to fosfomycin and colistin among CRE strains vary, with studies showing rates of 12.3–67.35% for fosfomycin and 9.1–76.19% for colistin. Notably, increasing colistin resistance has been highlighted in several reports (Qamar et al., 2017; Süzük Yıldız et al., 2019; El-Mahallawy et al., 2022). The optimal treatment strategy for CRE infections remains controversial; however, combination therapy has shown promise in significantly reducing morbidity and may represent the most effective approach for controlling these infections (Hu et al., 2022). To further refine treatment approaches, a Personalized Randomized Controlled Trial design has been introduced to evaluate strategies that balance efficacy and safety (Walker et al., 2021). These efforts, coupled with initiatives to mitigate the spread of resistant microbes and develop novel antibiotics or effective therapies, are critical for reducing mortality risk.

Patients with HD or those who have undergone HSCT are highly vulnerable to CRE infections, with mortality rates nearing 60%. Mortality from CRE bacteremia may be even higher due to delays of 2–3 days in initiating CRE-active therapy after bacteremia onset (Satlin et al., 2016). Our data indicate that patients undergoing HSCT, using central venous catheters, exposed to antibiotics (e.g., *β*-lactam/β-lactamase inhibitor combinations, echinocandins) in the past month, presenting with perianal skin ulceration in the past 3 days, or experiencing neutropenia ≥ 7 days, face an increased risk of CRE detection. These findings are consistent with previous reports (Ballo et al., 2019; Cao et al., 2022). Additionally, hospital LOS ≥ 18 days, recent steroid use (within 3 months), and albumin levels < 33.4 g/L were associated with higher CRE positivity, reflecting the complexity of HD patient management. This study highlights the need for vigilant monitoring of HD patients with these risk factors, along with implementing preventive measures such as reducing cross-transmission, improving hand hygiene, and optimizing antimicrobial stewardship.

Research on HD patients during the same period showed that 18.4% of patients colonized with CRE developed a subsequent CRE infection (Chen et al., 2023). Our data indicated a slightly higher rate, with 26.5% of the active screen-positive group experiencing a CRE infection in the later stages of their disease. This highlights the increased risk of subsequent infections in patients colonized with CRE. A study at an academic hospital in the southeastern United States implemented an active surveillance testing (AST) program, which improved compliance and increased the acceptance rate of CRE screening samples. Such programs enable timely isolation of CRE-positive patients, effectively preventing the spread of infection (Sova et al., 2021). Therefore, active CRE surveillance is a critical component of a comprehensive CRE prevention strategy, significantly reducing infection rates.

A study on AML patients found significantly reduced 60- and 90-day survival rates in those colonized with CRE compared to non-colonized patients (Ballo et al., 2019). Similarly, research in an ICU demonstrated that CRE colonization was associated with increased 90-day mortality, underscoring the adverse impact of CRE colonization on clinical outcomes (McConville et al., 2017). Consistent with these findings, our data showed a higher 90-day mortality rate in the detected CRE group compared to the undetected group. Independent risk factors for 90-day mortality included diarrhea within 3 days before CRE active surveillance and IL-6 levels ≥ 39.35 pg./mL within 24 h of surveillance. Among serum biomarkers, IL-6 not only showed the strongest response to early fever onset in HD patients, surpassing the composite index of CRP, IL-6, and PCT (Carcò et al., 2022), but was also closely associated with the occurrence of bacteremia (Lee et al., 2022).

The retrospective nature of our study presents inherent limitations. Despite adjusting for baseline differences such as hematological diseases and admission time, additional imbalances may still exist. Although PSM helped reduce confounding by measured covariates, it cannot account for unmeasured confounding, and the matched cohort may not fully represent the original study population, since patients without suitable matches are excluded. The modest sample size and limited number of outcome events resulted in wide confidence intervals for certain variables, notably diarrhea and elevated IL-6 levels, indicating potential instability and risks of overfitting or quasi-complete separation in our multivariate Cox regression analyses. Additionally, molecular confirmation methods, including PCR detection of carbapenemase genes, were not utilized. Therefore, our phenotypic-based definition might encompass non-carbapenemase-producing strains, potentially limiting the generalizability of our findings to settings routinely employing molecular diagnostics. Future larger multicenter studies employing molecular confirmation and more robust statistical methods such as penalized regression would enhance the precision and external validity of these findings. Nonetheless, our study provides valuable insights, suggesting that active CRE surveillance may be associated with 90-day mortality risk in hematology patients.

## 5 Conclusion

Our study highlights the elevated risk of CRE infection and mortality in the detected CRE group under active surveillance in the hematology department. Increasing the frequency of active screening significantly reduces infection rates. Additionally, active CRE surveillance may help identify patients at increased risk of 90-day mortality, based on observed associations in this study. Further studies are warranted to explore and refine proactive strategies for active CRE surveillance, aiming to better control CRE infection and transmission.

## Data Availability

The original contributions presented in the study are included in the article/Supplementary material, further inquiries can be directed to the corresponding authors.
