# Supplementary material for: Incidence and risk factors of active carbapenem-resistant enterobacteriaceae surveillance in hematology patients: a propensity score matching study
Source: Front Microbiol. 2025 Jul 16;16:1561587. doi: 10.3389/fmicb.2025.1561587 (PMC12307464; doi:10.3389/fmicb.2025.1561587)
Supplement: Supplementary file 2 [file Table_2.DOCX]

**Table S1. Distribution and composition rate of CRE strains.**

| **Species** | **Number of isolates (n=93)** | **Composition rate (%)** |
| --- | --- | --- |
| *Klebsiella pneumoniae* | 62 | 66.7 |
| *Escherichia coli* | 21 | 22.6 |
| *Enterobacter cloacae* | 6 | 6.5 |
| *Enterobacter albicans* | 1 | 1.1 |
| *Enterobacter aerogenes* | 1 | 1.1 |
| *Klebsiella oxytoca* | 1 | 1.1 |
| *Klebsiella ornithine solutes* | 1 | 1.1 |
