# Supplementary material for: Incidence and risk factors of active carbapenem-resistant enterobacteriaceae surveillance in hematology patients: a propensity score matching study
Source: Front Microbiol. 2025 Jul 16;16:1561587. doi: 10.3389/fmicb.2025.1561587 (PMC12307464; doi:10.3389/fmicb.2025.1561587)
Supplement: Supplementary file 3 [file Table_3.DOCX]

**Table S2. Univariate and multivariate logistic regression analysis of risk factors for isolation of CRE.**

| **Variable** | **Univariable model** | | **Multivariable model** | |
| --- | --- | --- | --- | --- |
|  | **OR (95%CI)** | **P value** | **OR (95%CI)** | **P value** |
| Receiving HSCT |  |  |  |  |
| No | 1.0 (Ref) |  | 1.0 (Ref) |  |
| Yes | 14.4(4.8-43.0) | <0.001 | 48.2 (4.9-474.8) | 0.001 |
| Hospital length of stays |  |  |  |  |
| <18 | 1.0 (Ref) |  | 1.0 (Ref) |  |
| ≥18 | 76.7 (24.6-239.1) | <0.001 | 23.7 (3.7-151.0) | 0.001 |
| Disease status |  |  |  |  |
| CR | 1.0 (Ref) |  | 1.0 (Ref) |  |
| NR | 3.9 (1.9-7.7) | <0.001 | 5.0(0.75-33.5) | 0.097 |
| Central venous catheter |  |  |  |  |
| Absence | 1.0 (Ref) |  | 1.0 (Ref) |  |
| Presence | 5.7 (2.5-12.9) | <0.001 | 20.8 (3.4-127.2) | 0.001 |
| Exposure to MDR infection within 3 months |  |  |  |  |
| No | 1.0 (Ref) |  | 1.0 (Ref) |  |
| Yes | 29.6(3.9-225.5) | 0.001 | 5.1 (0.1-254.2) | 0.417 |
| Exposure to steroid within 3 months |  |  |  |  |
| No | 1.0 (Ref) |  | 1.0 (Ref) |  |
| Yes | 11.1 (5.4-23.2) | <0.001 | 4.5 (1.1-18.3) | 0.037 |
| Exposure to antibiotics within last month |  |  |  |  |
| Fluoroquinolones |  |  |  |  |
| No | 1.0 (Ref) |  | 1.0 (Ref) |  |
| Yes | 5.2(2.2-12.9) | <0.001 | 1.1(0.109-11.1) | 0.937 |
| Aminoglycosides |  |  |  |  |
| No | 1.0 (Ref) |  | 1.0 (Ref) |  |
| Yes | 21.1(2.7-162.9) | 0.003 | 1.2(0.03-48.0) | 0.907 |
| Carbapenems |  |  |  |  |
| No | 1.0 (Ref) |  | 1.0 (Ref) |  |
| Yes | 6.5(2.5-16.8) | <0.001 | 3.1(0.26-37.3) | 0.374 |
| Polypeptide |  |  |  |  |
| No | 1.0 (Ref) |  | 1.0 (Ref) |  |
| Yes | 13.7 (4.6-41.0) | <0.001 | 1.8(0.14-22.06) | 0.656 |
| Triazole |  |  |  |  |
| No | 1.0 (Ref) |  | 1.0 (Ref) |  |
| Yes | 8.9 (4.3-18.3) | <0.001 | 0.28(0.05-1.53) | 0.141 |
| ß-lactam/ß-lactamase inhibitor |  |  |  |  |
| No | 1.0 (Ref) |  | 1.0 (Ref) |  |
| Yes | 32.0 (13.3-76.5) | <0.001 | 16.0 (2.9-88.1) | 0.001 |
| Echinocandins antifungal |  |  |  |  |
| No | 1.0 (Ref) |  | 1.0 (Ref) |  |
| Yes | 21.2 (7.1-63.3) | <0.001 | 12.0 (1.3-115.7) | 0.031 |
| Clinical symptoms |  |  |  |  |
| Oral ulcer |  |  |  |  |
| No | 1.0 (Ref) |  | 1.0 (Ref) |  |
| Yes | 5.0(1.4-18.1) | 0.016 | 0.21(0.01-3.52) | 0.276 |
| Cough |  |  |  |  |
| No | 1.0 (Ref) |  | 1.0 (Ref) |  |
| Yes | 3.1(1.5-6.4) | 0.002 | 2.3(0.15-34.6) | 0.546 |
| Expectoration |  |  |  |  |
| No | 1.0 (Ref) |  | 1.0 (Ref) |  |
| Yes | 2.8(1.2-6.5) | 0.018 | 0.80(0.04-17.15) | 0.888 |
| Diarrhea |  |  |  |  |
| No | 1.0 (Ref) |  | 1.0 (Ref) |  |
| Yes | 7.5 (1.6-34.5) | 0.009 | 3.54(0.12-106.6) | 0.466 |
| Perianal skin ulceration |  |  |  |  |
| No | 1.0 (Ref) |  | 1.0 (Ref) |  |
| Yes | 10.4 (4.9-25.6) | <0.001 | 7.3 (3.8-16.4) | <0.001 |
| Laboratory examination within 24h |  |  |  |  |
| Body temperature |  |  |  |  |
| <37.3 | 1.0 (Ref) |  | 1.0 (Ref) |  |
| ≥37.3 | 3.2 (2.1-5.1) | <0.001 | 2.57(0.49-13.3) | 0.262 |
| Presence of neutropenia |  |  |  |  |
| No | 1.0 (Ref) |  | 1.0 (Ref) |  |
| Yes | 3.6 (1.2-7.2) | <0.001 | 0.92(0.19-4.5) | 0.915 |
| Albumin, g/L |  |  |  |  |
| ≥33.4 | 1.0 (Ref) |  | 1.0 (Ref) |  |
| <33.4 | 8.2 (3.9-17.1) | <0.001 | 6.0 (1.1-33.0) | 0.039 |
| Duration of neutropenia prior |  |  |  |  |
| to CRE active surveillance |  |  |  |  |
| <7 | 1.0 (Ref) |  | 1.0 (Ref) |  |
| ≥7 | 19.3 (6.5-57.5) | <0.001 | 38.5 (3.0-493.9) | 0.005 |

Abbreviations: HSCT, hematopoietic stem cell transplantation; CRE, carbapenem-resistant Enterobacteriaceae; CR, complete remission; NR non-remission; MDR, multiple drug resistance.
