# Supplementary material for: Incidence and risk factors of active carbapenem-resistant enterobacteriaceae surveillance in hematology patients: a propensity score matching study
Source: Front Microbiol. 2025 Jul 16;16:1561587. doi: 10.3389/fmicb.2025.1561587 (PMC12307464; doi:10.3389/fmicb.2025.1561587)
Supplement: Supplementary file 4 [file Table_4.DOCX]

**Table S3. Univariate and multivariate cox regression analysis of risk factors associated with 90-day mortality among patients detected CRE.**

| **Variable** | **Univariable model** | | **Multivariable model** | |
| --- | --- | --- | --- | --- |
|  | **HR (95%CI)** | **P value** | **HR (95%CI)** | **P value** |
| Age, years |  |  |  |  |
| <60 | 1.00 (Ref) |  | 1.00 (Ref) |  |
| ≧60 | 1.985(1.071-3.681) | 0.030 | 2.548(0.569-11.409) | 0.222 |
| Receiving HSCT |  |  |  |  |
| No | 1.00 (Ref) |  | 1.00 (Ref) |  |
| Yes | 0.439(0.231-0.834) | 0.012 | 2.639(0.271-25.721) | 0.404 |
| Hematological Disease status |  |  |  |  |
| CR | 1.00 (Ref) |  | 1.00 (Ref) |  |
| NR | 3.580(1.414-9.065) | 0.007 | 6.592(0.541-80.401) | 0.139 |
| Central venous catheter |  |  |  |  |
| Absence | 1.00 (Ref) |  | 1.00 (Ref) |  |
| Presence | 0.460(0.214-0.988) | 0.046 | 0.073(0.004-1.196) | 0.067 |
| **Exposure to antibiotics, within 1 months** |  |  |  |  |
| Fluoroquinolones |  |  |  |  |
| No | 1.00 (Ref) |  | 1.00 (Ref) |  |
| Yes | 2.267(1.223-4.202) | 0.009 | 0.867(0.166-4.521) | 0.866 |
| ß-lactam/ß-lactamase inhibitor |  |  |  |  |
| No | 1.00 (Ref) |  | 1.00 (Ref) |  |
| Yes | 3.276(1.465-7.329) | 0.004 | 0.529(0.021-13.146) | 0.698 |
| Polypeptide |  |  |  |  |
| No | 1.00 (Ref) |  | 1.00 (Ref) |  |
| Yes | 2.812(1.574-5.027) | <0.001 | 3.018(0.644-14.141) | 0.161 |
| Triazole antifungal |  |  |  |  |
| No | 1.00 (Ref) |  | 1.00 (Ref) |  |
| Yes | 2.331(1.295-4.195) | 0.005 | 1.037(0.185-5.793) | 0.967 |
| **Clinical symptoms** |  |  |  |  |
| Cough |  |  |  |  |
| No | 1.00 (Ref) |  | 1.00 (Ref) |  |
| Yes | 2.233(1.246-4.002) | 0.007 | 1.484(0.095-23.248) | 0.779 |
| Expectoration |  |  |  |  |
| No | 1.00 (Ref) |  | 1.00 (Ref) |  |
| Yes | 2.133(1.151-3.953) | 0.016 | 0.931(0.070-12.435) | 0.957 |
| Abdominal pain |  |  |  |  |
| No | 1.00 (Ref) |  | 1.00 (Ref) |  |
| Yes | 2.988(1.332-6.699) | 0.008 | 1.879(0.057-61.882) | 0.723 |
| Diarrhea |  |  |  |  |
| No | 1.00 (Ref) |  | 1.00 (Ref) |  |
| Yes | 2.785(1.412-5.492) | 0.003 | 9.735(1.159-81.778) | 0.036 |
| **Clinical laboratory test results within 24 hours of CRE active surveillance** |  |  |  |  |
| Albumin, g/L |  |  |  |  |
| ≧37.9 | 1.00 (Ref) |  | 1.00 (Ref) |  |
| <37.9 | 2.550(1.189-5.467) | 0.016 | 0.723(0.094-5.535) | 0.755 |
| C reactive protein, mg/L |  |  |  |  |
| <36.82 | 1.00 (Ref) |  | 1.00 (Ref) |  |
| ≧36.82 | 4.53(1.993-10.298) | <0.001 | 2.050(0.275-15.305) | 0.484 |
| Interleukin-6, pg/ml |  |  |  |  |
| <39.35 | 1.00 (Ref) |  | 1.00 (Ref) |  |
| ≧39.35 | 2.570(1.206-5.476) | 0.014 | 7.762(1.293-46.594) | 0.025 |
| Body temperature |  |  |  |  |
| <37.3 | 1.00 (Ref) |  | 1.00 (Ref) |  |
| ≧37.3 | 2.557(1.393-4.695) | 0.002 | 2.759(0.292-26.065) | 0.376 |

Abbreviations: CRE, carbapenem-resistant Enterobacteriaceae; HSCT, hematopoietic stem cell transplantation.
